# *MafA* Overexpression: A New Efficient Protocol for *In Vitro* Differentiation of Adipose-Derived Mesenchymal Stem Cells into Functional Insulin-Producing Cells

**DOI:** 10.22074/cellj.2019.5669

**Published:** 2019-02-25

**Authors:** Dian Dayer, Mohammad Reza Tabandeh, Eskandar Moghimipour, Mahmood Hashemi Tabar, Ata Ghadiri, Elham Allah Bakhshi, Mahmoud Orazizadeh, Mohammad Ali Ghafari

**Affiliations:** 1 Cellular and Molecular Research Center, Ahvaz Jundishapur University of Medical Sciences, Ahvaz, Iran; 2 Department of Biochemistry and Molecular Biology, Faculty of Veterinary Medicine, Shahid Chamran University of Ahvaz, Ahvaz, Iran; 3 Stem Cells and Transgenic Technology Research Center, Shahid Chamran University of Ahvaz, Ahvaz, Iran; 4 Department of Pharmaceutics, Faculty of Pharmacy, Ahvaz Jundishapur University of Medical Sciences, Ahvaz, Iran; 5 Department of Anatomy, Faculty of Medicine, Ahvaz Jundishapur University of Medical Sciences, Ahvaz, Iran; 6 Department of Immunology, Faculty of Medicine, Ahvaz Jundishapur University of Medical Sciences, Ahvaz, Iran; 7 Department of Biochemistry, Faculty of Medicine, Ahvaz Jundishapur University of Medical Sciences, Ahvaz, Iran

**Keywords:** Adipose Tissue, Insulin-Producing Cells, *MafA*, Mesenchymal Stem Cells

## Abstract

**Objective:**

We proposed a novel differentiation method for the efficient differentiation of adipose-derived mesenchymal stem cells (ADMSCs) into functional insulin-producing cells (IPCs) based on *MafA* overexpression.

**Materials and Methods:**

In this experimental study, a eukaryotic expression vector containing *MafA [MafA/pcDNA3.1(+)]* was constructed and purified. ADMSCs were differentiated into IPCs. ADMSCs were assigned in two groups including control (C), and the *MafA* overexpressed (*MafA*+) groups. The ADMSCs were transfected by *MafA/pcDNA 3.1(+)* at day 10 of the differentiation. Differentiated cells were analyzed for the expression of multiple β cell specific genes (*Nkx2.2, Ngn3, Isl-1, Pdx1, MafA, Nkx6.1,* and *Insulin*) using real-time polymerase chain reaction (PCR). The insulin secretion potency of the differentiated cells in response to glucose exposure was also determined using an enzyme-linked immunosorbent assay (ELISA) method and Dithizone (DTZ) staining. The IPCs from the control manipulated group, and un-differentiated ADMSCs group were transplanted to streptozotocin (STZ)-diabetic rats. Rats were monitored for blood glucose and insulin concentration.

**Results:**

The results revealed that ADMSCs were successfully differentiated into IPCs through the 14 day differentiation protocol. The expression of β-cell specific genes in *MafA*+ IPCs was higher than in control cells. Glucose-induced insulin secretion after the exposure of IPCs to glucose was higher in *MafA*+ group than the control group. The STZ- diabetic rats showed an ability to secrete insulin and apparent hyperglycemic condition adjustment after transplantation of the control IPCs. The mean insulin concentration of diabetic rats that were transplanted by manipulated IPCs was significantly higher than ADMSCs-transplanted rats; however, no effect was observed in the concentration of blood glucose.

**Conclusion:**

The overexpression of *MafA* can be used as a novel promising approach for the efficient production of IPCs from ADMSCs *in vitro*. However, the future therapeutic use of the *MafA*+ IPCs in diabetic animals needs further investigations.

## Introduction

Diabetes mellitus is the most common metabolic disorder worldwide. With regard to the significant increase in the number of diabetic patients and diabetic complications, much of the latest scientific research is focused on the design of a reliable plan for the treatment of diabetes mellitus (1). In diabetes mellitus type 1 (T1DM) the autoimmune destruction of pancreatic beta cells results in insufficient insulin secretion (2). Stem cell therapy can be regarded as one of the most interesting methods of the production of functional pancreatic beta cells (3). Some limitations of this approach are the generation of cells with immature or abnormal appearance and the lack of insulin secretion ability (4). In this view, the optimization of differentiation protocols is inevitable. Recently, several genetic manipulations have been developed in order to generate the functional artificial pancreatic beta cells (5-7). *MafA* is a transcription factor with a b-zip design which belongs to *MafA* family. MafA protein binds to the insulin enhancer element, RIPE3b, of the insulin gene promoter and activates the insulin gene expression (8). 

Synergistic cooperation of *MafA* with NeuroD and Pdx1 increases the insulin synthesis and secretion. Moreover, *MafA* coordinates with MafB to induce pancreatic ß cells generation and differentiation (9). *MafA* regulates the glucose and energy balance in different tissues such as adipose tissue, pancreas, and muscle, and its deficiency in mice leads to diabetes and diabetic nephropathy (10). Some studies emphasized the eventual role of *MafA* in the differentiation of adipocytes and adipose tissue sensitivity to insulin (11-13). Given these findings, it has been suggested that *MafA* can be used as an effective factor for the renewal of pancreatic ß cells and the induction of differentiation of stem cells into insulin-producing cells (IPCs) (14). The study by Chiou et al. (15) showed that *MafA* promotes the reprogramming of placenta-derived multipotent stem cells into pancreatic islets-like cells. With regard to the significant role of *MafA* in the production and maintenance of mature beta cells, we designed a novel protocol for the differentiation of adipose-derived mesenchymal stem cells (ADMSc) into functional IPCs by the overexpression of *MafA*.

## Materials and Methods

### Cloning of *MafA* into a pcDNA3.1+ plasmid vector

In this experimental study, the RNX^TM^ reagent (Sinaclon, Iran) was used for the isolation of the total RNA as recommended by the manufacturer. The purity of isolated RNA was assessed using a Nanodrop spectrophotometer (Nanodrop 2000^TM^, Thermo, Canada). The reaction of cDNA synthesis was carried out using a CycleScript RT PreMix cDNA synthesis kit (Bioneer, South Korea) in a total volume of 20 µL according to the manufacturer’s recommendation. The PCR reaction was performed utilizing Taq DNA Polymerase 2X Master Mix Red (Ampliqon, Denmark) in a total amount of 20 µL. Mgcl_2_ and each of the primer concentrations were modified to 1.5 mM and 250 nM, respectively. The primers (Bioneer, South Korea) which were designed for the generation of full-length *MafA* gene were as follow: 

5´-ATATAAGCTTAATATGGCCGCGGAGCTGGC3 ´and 5´-ATCGGGATCCTCACAGAAAGAAGTCG-3´.

The Primer Premier 5 software (Premier Biosoft International, USA) was used for the design of particular primers with restriction sites at the 5´ (HindIII) (Vivantis Malaysia) and 3´ends (EcoRI) (Vivantis Malaysia). Polymerase chain reaction (PCR) was performed using a Thermal Cycler (Eppendorf Mastercycler, Germany). The thermal cycle included 35 cycles as follows: 5 minutes at 95°C for the initial denaturation, 1 minute at 94°C for denaturation, 1 minute at 58°C for annealing, 1 minute at 72°C for the extension and a final extension at 72°C for 5 minutes. The amplified PCR products were visualized by 1% agarose gel electrophoresis in TAE buffer stained with DNA Safe stain (Merck, Germany) under ultraviolet (UV) light (Mabna Tajhiz, Iran). The *MafA* PCR product was purified from the agarose gel using a Gel DNA Recovery Kit (SinaClon BioSciences, Iran) according to the manufacturer’s recommendation. Double digestion of PCR products and pcDNA3.1+ vector (ThermoFisher Scientific, USA) were performed utilizing EcoRI and the Hind III restriction enzymes at 37°C for 2 hours. The digested fragments were visualized using agarose gel electrophoresis. The fragments were purified by a Gel DNA Recovery Kit (Bioneer, South Korea) according to the manufacturer’s recommendation. The obtained purification linear vector and insert were ligated to each other using T4 DNA ligase (Fermentas, USA). The reaction was deactivated by the incubation for 15 minutes at 65°C. The competent cells were prepared from E. coli Top10F' cell (Clontech Laboratories, Inc USA) using the calcium chloride method. The obtained competent cells were transformed with 2 µL of the ligation product. The positive transformed bacterial cells were picked up on LB medium agar plates containing ampicillin (100 µg/ ml, Sigma, USA). Some of the colonies were confirmed by colony PCR using universal T7 and BGH primers (Bioneer, South Korea). After the selection of the positive recombinant clones, the plasmid DNA was extracted from the cells cultured overnight using a Miniprep plasmid isolation kit (SinaClon, Biosciences, Iran) and confirmed by PCR, restriction enzyme digestion, followed by DNA sequencing using T7 and BGH primers. The plasmid was purified using an AccuPrep Nano Plus Plasmid Mini Extraction Kit (Bioneer, Korea) and sequenced using a Big Dye terminator V.3.1 Cycle Sequencing Kit in an ABI 3130 Genetic analyzer (Applied Biosystems, USA).

### Preparation of tissues

Normal Sprague Dawley male rats (n=5) with an age range of 2-3 months were chosen for the experiment. All animals used were housed in accordance to the Guide for the Care and Use of Laboratory Animals by the National Academy of Sciences (National Institutes of Health Publication No. 86-23). The animal experiment was approved by the Animal Experiments Committee of the Ahvaz Jundishapur University of Medical Sciences (AJUMS.REC.1393.100). Rats were anesthetized with a mixture of 100 mg/kg ketamine (Sigma, USA) and 10 mg/kg xylazine (Sigma, USA). Pancreatic tissue and adipose tissue from splanchnic region isolated in a sterile condition. The tissues were washed three times with sterile PBS that contained 3% Pen /Strep (Gibco, UK).

### Isolation of rat of adipose-derived mesenchymal stem cells 

The isolated splanchnic adipose tissue was chopped into very small pieces. The explants were placed in the 25 cm^2^ culture flask. Three milliliters of Dulbecco’s Modified Eagle’s Medium-high glucose (DMEM-HG, Gibco, Netherlands) containing 15% fasting blood glucose (FBS, Sigma, USA) and 1% Pen/Strep was gently added to each flask. Flasks were placed in a 37°C incubator with 5% CO_2_. After 4 days, the culture medium was replaced by DMEM-HG containing 10% FBS and 1% Pen/Strep. When the adherent cells reached confluence, the explants were removed. The culture medium was replaced every 3 days. 

### Characterization of adipose-derived mesenchymal stem cells

The expression of cell surface biomarkers named clusters of differentiation (CD) including CD34, CD45, CD90, and CD105 was determined using flow cytometry method, as described previously. The osteogenic and adipogenic differentiation potency of ADMSCs were assessed using the osteogenic and adipogenic mediums, as described previously (16, 17).

### The protocol for differentiation of adipose-derived mesenchymal stem cells into insulin producing cells

The isolated cells were pooled, counted, and randomly divided into 2 groups based on the modification of the basic differentiation protocol. The experimental groups included the control group and the *MafA* overexpressed (*MafA*+) groups. All experiments were done in triplicates (three flasks for each differentiation protocol). In the control group, the basic differentiation protocol was performed. The basic differentiation protocol consisted of 3 main stages. In stage 1, cells (1×10^6^/ml) were cultured in a medium containing DMEM-LG (Gibco, Netherland), 10% FBS, and 1% Pen/Strep until the cells reached 80% confluency. In stage 2, the differentiation medium contained DMEM-low glucose (DMEM-LG), 20 µM nicotinamide (Sigma, USA), 5% FBS, and 1% Pen/Strep for 7 days. In stage 3, cells were cultured in a medium of stage 2 plus 10 µM Exendix-4 (Sigma, USA) for 7 days (16). In the *MafA*+ group, cells were differentiated through basic differentiation protocol, and then, transfected with a recombinant *MafA*/ pCDNA3.1(+) vector at day 3 of stage 3.

### Transfection of differentiated adipose-derived mesenchymal stem cells by the recombinant vector

Differentiated ADMSCs were trypsinized and seeded in 25 ml flasks 24 hours before the transfection. At day 10 of differentiation, cells were washed three times with phosphate buffered saline (PBS, Calbiochem, Iran), trypsinized, counted, and suspended at a density of 106/ml in serum-free DMEM-HG. Then, 100 µl of cells were mixed with 5 µg of suitable vector pCDNA(3.1+) in control group and recombinant *MafA*/pCDNA 3.1(+) in the experimental group in 0.4 ml electroporation cuvette (Biorad, USA), and gently mixed by pipetting. The mixture was placed in an electroporation system (GenePulser system II, Biorad, USA) and one pulse of 140V was delivered for 15 milliseconds. Following the electroporation, cells were plated onto a 25 ml flask that contained a differentiation medium and was incubated at 37°C and 5% CO_2_. After 24 hours, Genticin (350 µg/ml, Sigma, USA) was added to the growth media for the positive selection of antibiotic-resistant ADMSCs. The media were changed every 3 days, and Genticin selection was maintained for 7 days. Antibiotic-resistant ADMSCs were split to be grown for the differentiation.

### Detection of *MafA* expression in transfected cells

In order to confirm the overexpression of *MafA* in *MafA/pCDNA 3.1(+)* transfected cells, dot blot analysis, indirect enzyme-linked immunosorbent assay (ELISA), and real-time PCR were performed. Genticin resistant ADMSCs were split to grow for 24 hours at 37°C. Cells were trypsinized and centrifuged at 1200 ×rpm for 8 minutes; then washed with PBS. Cell lysis was done using radio immune precipitation assay (RIPA) buffer consisted of 50 mM HCl, 150 mM NaCl, 0.1% Triton X-100, 0.1% sodium dodecyl sulfate (SDS), 1mM EDTA, 1 mM NaF, and 1 mM phenyl methyl sulfonyl fluoride (PMSF) in ddH_2_O. Samples were centrifuged at 10000 × rpm for 10 minutes, and the supernatants were separated for further analysis. Protein concentration was determined by the Bradford method using 1 mg/ml bovine serum albumin as a standard.

### Dot blot analysis 

A nitrocellulose membrane (Millipore, USA) was prewetted for 5 minutes in a mixture of tris-buffered saline and Tween 20 (TBS-T) (20 mM Tris, 150 mM NaCl, 0.05% Tween 20, pH=7.5), and then, soaked in distilled water for 2 minutes. The lysate of control and *MafA/pCDNA 3.1(+)* transfected cells (~10 ug protein) was dotted on nitrocellulose membrane. Non-specific binding sites were then blocked using TBS-T containing 5% skim milk (Merck, Germany) for 30 minutes at room temperature, rinsed three times with TBS-T, and incubated for 30 minutes with 1:1000 dilution of specific antibody against rat *MafA* protein (Santa Cruz Biotechnology, USA, Art No:sc-390491). This antibody was a mouse monoclonal antibody specific for an epitope mapping between amino acids 330-341 which are near the C-terminus of *MafA*, recommended for the detection of *MafA* of the mouse, rat, and human origin. The membrane was incubated for 30 minutes with rabbit anti-rat HRP-conjugated IgG antibody (Santa Cruz Biotechnology, USA, Art No: SC2786) with a dilution of 1:1000. Following three washes with PBS buffer, the substrate [50 mM Tris buffer, pH=7.8, containing 6 mg 3’-Diaminobenzidine (DAB), 10 uL H_2_O_2_] was used for the detection. 

### Indirect ELISA for the detection of *MafA* expression in transfected cells

Microwell plates (Nunc, Denmark) were coated with 100 µl per well of the *MafA* antibody (Santa Cruz Biotechnology, USA, Art No: sc-390491) (500 ng), diluted in coating buffer (0.2 M sodium carbonate/ bicarbonate, pH=9.4), and incubated overnight at room temperature. After washing the plates three times with PBST (PBS with 0.05% v/v Tween 20), the unbound sites were blocked with 200 µl of blocking solution at 37°C for 1 hour. Then, the plates were washed three times with PBST. After that, 100 µl of cell lysates were added into each well and incubated at room temperature for 1 hour. After the plates were washed three times with washing solution, 100 µl of the *MafA* antibody (500 ng) diluted in coating buffer was pipetted into each well and incubated for 1 hour at room temperature. Then, the plates were washed three times with washing buffer and 100 µl of rabbit anti-rat horseradish peroxidase (HRP)-conjugated IgG antibody (Santa Cruz Biotechnology, USA, Art No: SC-2786, 1:1000) diluted in PBST was added and incubated for 1 hour at room temperature. The plates were washed five times, and 150 µl of substrate solution (0.1 mg/ ml 3,3´,5,5´-Tetramethylbenzidine (TMB) in 0.1 M citrate- phosphate buffer, pH=5.0 containing 0.03% hydrogen peroxide) was added into each well. The reaction was stopped after 30 minutes by adding 50 µl of 1.25 M sulfuric acid, and the absorbance was read in a microplate reader (BioTek, USA) in a dual wavelength mode (450-630 nm). Lysis buffer was used as a blank control. The validation of the antibody was performed using mouse eye extract as a positive control (Santa Cruz Biotechnology, USA, Art No: sc-364241). All assays were performed in triplicate. Data was reported by the unit of OD450 nm/ mg protein.

### Real time polymerase chain reaction

The gene expression pattern between the control and experimental groups was compared. The details are available in "the evaluation of IPCs functionality in vitro" section.

### The evaluation of insulin producing cells functionality *in vitro*

#### Dithizone staining

At the end of the differentiation protocol, DTZ (Sigma Aldrich, USA) solution (100 ng/ml) was dissolved in dimethyl sulfoxide (Sigma Aldrich, USA). After filtrating through a 0.2 µm filter, DTZ solution was added to each 25 cm^2^ flask at the volume of 3 ml. Cells were incubated at 37°C for 30 minutes and washed three times with PBS. Cells were analyzed using an inverted microscope (Olympus, Japan) for the detection of Crimson red-stained clusters.

### Real-time polymerase chain reaction analysis

At the end of the experiment, the two obtained groups of differentiated cells were analyzed for the gene expression through real-time PCR. At day 14 of differentiation, differentiated cells isolated. The RNA extraction was performed by the use of RNX^TM^ reagent (CinaClon, Iran) according to the manufacturer’s recommendation. One µg of produced RNA was used for cDNA synthesis by utilization of a CycleScript cDNA synthesis kit (CycleScript RT PreMix Bioneer, South Korea) based on the manufacturer’s recommendation. The real-time PCR reaction was carried out by means of an Ampliqon RealQ Plus Master kit for SYBR Green I^®^ (Ampliqon, Denmark) on a Lightcycler^®^ Detection System (Roche, USA), as described previously (17, 18). Table 1 shows the list of the genes and primers used for real-time PCR. The negative controls consisted of two distinct reactions without cDNA or RNA. The 2^-ΔΔCt^ method was performed to compare the gene expression between different groups (19). All qPCR analyses were performed according to the Minimum Information for Publication of Quantitative Real-Time PCR Experiments (MIQE) guideline (20). 

**Table 1:** Characteristics of primers used in real-time polymerase chain reaction


Gene name	Sequence (5́´-3́´)	length (bp)	Accession number

*GAPDH*	F: TG GTATCGTGGAAGGACTC	290	NM_002046/6
	R: CCTGCTTCACCACCTTCTTG		
*Pdx1*	F: GGAGGGTTTGGAAAACCAGT	131	NM_022852.3
	R: ACAAACATAACCCGAGCACA		
*Nkx2.2*	F: AAACCGTCCCAGCGTTAAT	126	NM_001191904.1
	R: TGCTTTAGAAGACGGCTGAC		
*Nkx6.1*	F: ACACACGAGACCCACTTTTT	147	NNM_031737.1
	R: TTCTGGAACCAGACCTTGAC		
*Isl-1*	F: GCTTTTCAGCAACTGGTCA	123	NM_017339.3
	R: AATAGGACTGGCTACCATGC		
*Insulin*	F: ATCTTCAGACCTTGGCACTG	141	NM_019129.3
	R: ATCTTCAGACCTTGGCACTG		
*MafA*	F: CTGCTGTCCTACTATGCTCA	137	XM_006241903.2
	R: TGTATTTCCCCAGGAGTTACAG		
*Ngn-3*	F: CTATTCTTTTGCGCCGGTAC	128	NM_021700.1
	R: CTGACGGTCACTTGGCAG		


### Insulin secretion assay 

The ability of different IPCs for the synthesize and secretion of insulin was compared through the insulin secretion assay, as described previously. The insulin concentration was determined using a rat-specific insulin ELISA kit (RayBiotech, USA) based on a protocol recommended by the manufacturer, as described previously (17). The concentration of insulin was reported as µIU/ml. 

### Transplantation of insulin-producing cells and evaluation of insulin-producing cells functionality *in vivo*

The study group consisted of 15 normal male Sprague Dawley rats with 8 weeks age and 180-200 g weight. The experimental diabetes mellitus condition was induced using 50 mg/kg of Streptozotocin (STZ, Sigma Aldrich, USA) in citrate buffer. Rats possessed three blood glucose above 500 mg/ml (at least three measurements) were chosen as diabetic. The diabetic rats were studied in three groups. Group 1 (n=5) was injected with undifferentiated ADMSCs, the control group (n=5) received un-manipulated IPCs. The remained group (n=5) was injected with manipulated IPCs. At day 14 of differentiation, the differentiated IPCs of all three experimental groups were detached by trypsinization. After washing three times with PBS, 1×10^6^ of isolated cells were suspended in 200 µl of DMEM-HG. Rats were anesthetized using 100 mg/kg ketamine and 10 mg/kg xylazine as a mixture. The differentiated cells were injected through the tail vein into rats (19). The blood glucose concentration was determined once a week by utilizing a glucometer (EasyGluco, South Korea). After six weeks, a 25 mM glucose solution was injected into rats. After 10 minutes, rats were anesthetized using 100 mg/Kg ketamine and 10 mg/Kg xylazine as a mixture, and 2 ml of whole blood was acquired. After 5 minutes, the serum was obtained by centrifugation at 2000 rpm. The insulin concentration in serums was measured by an ELISA method. 

### Statistical analyses

Data analyses were done using the SPSS 18.0 software package (SPSS Inc., Chicago, IL, USA). All analyses were done in triplicate. One-way ANOVA followed by Tukey post-hoc analysis was used to test differences between various means including the expression level of different genes and insulin concentration. The difference between two independent groups was determined using t test. All experimental data were presented as the mean ± SEM. The level of significance for all tests was set at P<0.05.

## Results

### Characteristics of *MafA*-pCDNA3.1(+) vector

**Fig.1:**
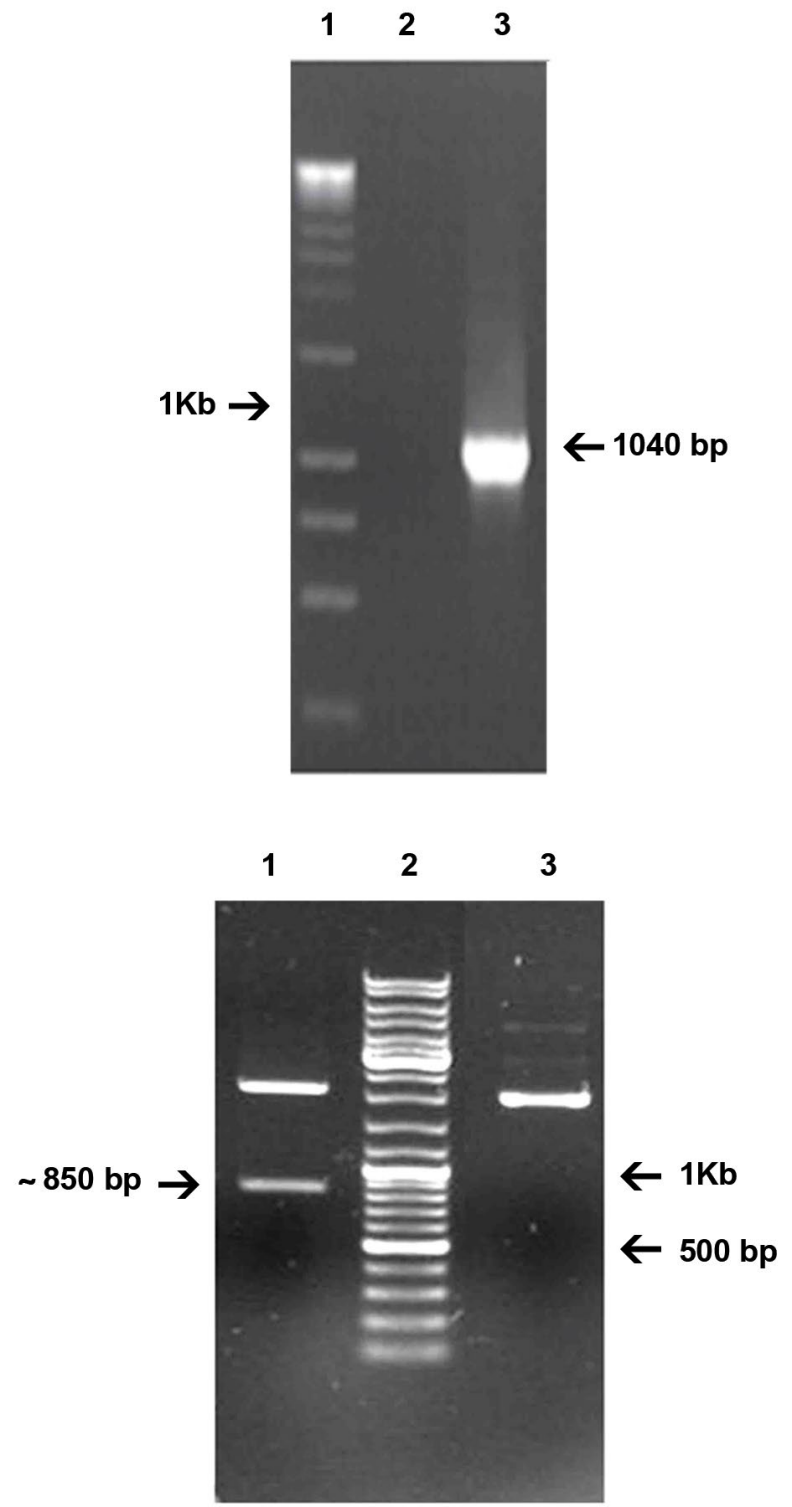
Polymerase chain reaction screening of positive clone of *E.coli* Top10F’ containing MafA/pCDNA3.1(+) vector using universal primer (T7 promoter and BGH reverse primers) on 1% agarose gel electrophoresis. **A.** Lane 1; 1 kb DNA ladder, Lane 2; Negative control, and Lane 3; A 1040 bp band corresponding to 870 bp *MafA* gene and 170 bp flanking regions of plasmid and **B.** The analysis of the enzyme digestion for the recombinant MafA/pCDNA 3.1(+) vector. Lane 1; A 870 bp *MafA* gene separated from the recombinant vector after digestion using EcoRI and HindIII enzymes, Lane 2; 100 bp DNA ladder, and Lane 3; Recombinant MafA/pCDNA 3.1(+) before the digestion.

**Fig.2:**
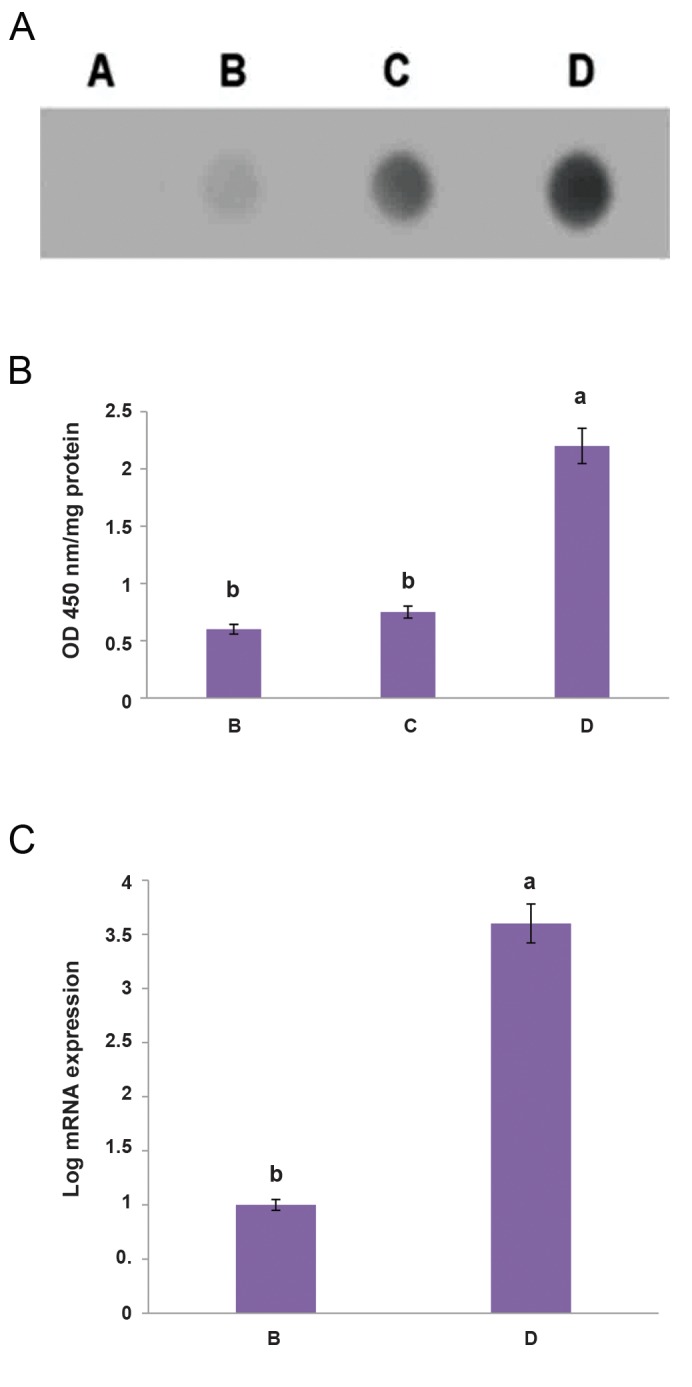
Protein and mRNA expression analysis of *MafA* in transfected adipose- derived mesenchymal stem cells (ADMScs). **A.** Dot blot, **B.** ELISA, and **C.** Real- time polymerase chain reaction (PCR) analysis of *MafA* expression in ADMSCs after the transfection with pCDNA 3.1+ or MafA/pCDNA 3.1(+) plasmids. A. Blank (cell lysis buffer), B. ADMSCs transfected with pCDNA 3.1+, C. Mouse eye extract (positive control) (Santa Cruz Biotechnology, USA, Art No: sc364241), and D. ADMSCs transfected with *MafA/pCDNA 3.1(+)* recombinant vector. GAPDH was used as a calibrator for real-time PCR analysis. Data are expressed as the mean ± SE. The statistical significance difference at P<0.05 is represented by different letters.

According to the applied primers in RT-PCR step, colony PCR, restriction site digestion, and DNA sequencing, the accuracy of *MafA* cloning into pCDNA3.1(+) plasmid was confirmed (Fig .1). Dot blot analysis, ELISA, and Real-time quantitative PCR were performed on selected Genticin resistant ADMSCs clones in order to determine the expression of *MafA* in transfected cells. Dot blot results showed a low level of *MafA* protein in ADMSCs cells transfected with pCDNA3.1(+) and a high level of *MafA* protein in *MafA/ pCDNA 3.1(+) *transfected cells (Fig .2A). The ELISA and Real-time PCR analysis of Genticin resistant ADMSCs clones showed a significant increase in *MafA* expression compared with the control cells (Fig .2B, C). 

### Characterization of adipose-derived mesenchymal stem cells

*In vitro* differentiation of adipose-derived mesenchymal stem cells into adipocytes and osteocytes

In order to confirm the multipotent ability of ADMSCs, after the third passage, cells were cultured in the adipogenic or osteogenic mediums. The results confirmed the differentiation of ADMSc into osteocytes and adipocytes. The deposits of calcium were visualized by Alizarin red staining showing the osteocytes formation (results are not shown). The vacuoles of lipids were also exhibited by oil red o staining identified the adipocytes formation (17) (results are not shown). 

### The identification of adipose derived mesenchymal stem cells surface glycoproteins 

ADMSCs were evaluated for the expression of specialized surface cell markers of mesenchymal stem cells by flow cytometry. The results showed 99% positive expression of CD90 and 98% positive expression of CD105. ADMSCs were negative for CD34 and CD45 antigens (17) (results are not shown). 

### Evaluation of differentiation stages

#### The morphology of differentiated cells 

**Fig.3:**
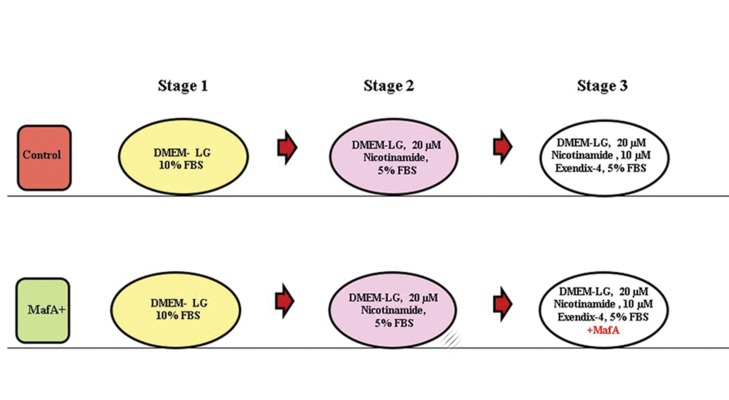
Differentiation protocol of ADMSCs into IPCs. ADMSCs in control group were differentiated into IPCs using the three stages basic protocol. Cells in MafA+ group were transfected with MafA/pCDNA 3.1(+) recombinant plasmid. Undifferentiated ADMSCs showed spindle- like shape at the beginning of differentiation. The number of cells with epithelial-like shape was increased at day 7 of differentiation. IPCs that were distinctly stained as crimson red with DTZ became apparent at the final step of differentiation. ADMSCs; Adipose derived mesenchymal stem cells, IPCs; Insulin producing cells, DTZ; Dithizone, and DMEM-LG; Dulbecco’s Modified Eagle’s Medium-low glucose.

In passage 3, all ADMSCs were mesenchymal stem cells with the fibroblast-like shape. Changes in ADMSCs appearance during the 14 days of differentiation are shown in Figure 3. Spindle-like ADMSCs were gently changed to round epithelial-like cells during the stage 2 of differentiation. By the progression of differentiation, cells began to shortening slowly and were gathered together. In stage 3 of differentiation, the morphology of cells changed to spheroid-like shape with similarity to pancreatic islets. The differentiated cells were stained as Crimson red with DTZ (Fig .3). 

### Evaluation of insulin-producing cells functionality *in vitro*

#### The expression of critical pancreas-related genes after the *MafA* overexpression

**Fig.4:**
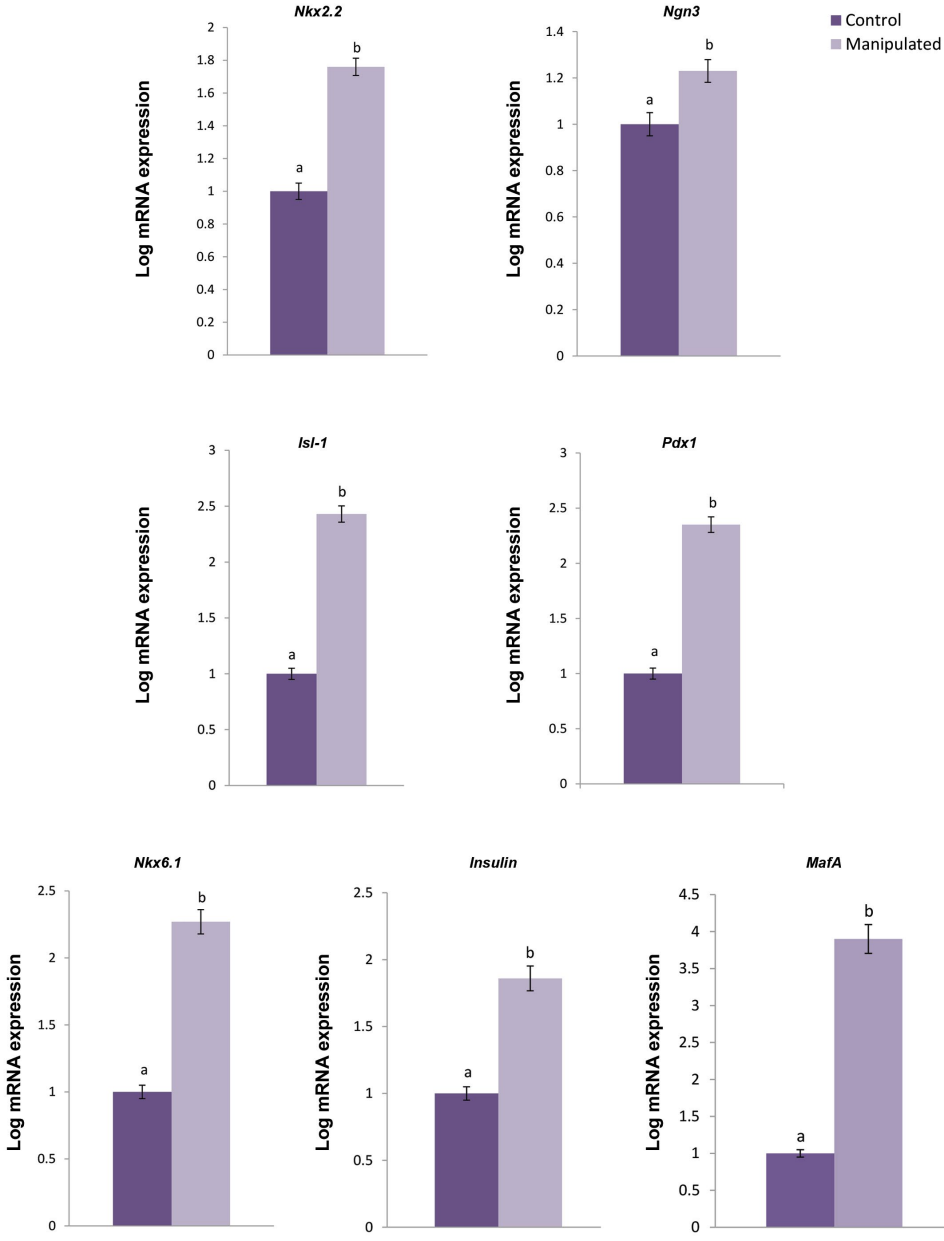
The expression of pancreas-related genes after the *MafA* overexpression. The over-expression of *MafA* had high stimulatory effect on the expression of Pdx1, *MafA, Nkx2.2, Nkx6.1, Ngn3, Isl1,* and Insulin (P<0.05). GAPDH was used as a calibrator for real-time polymerase chain reaction (PCR) analysis. Data are expressed as the mean ± SE. The statistical significance difference at P<0.05 is represented by different letters.

Comparison between the different groups of differentiated cells in the expression of specific genes involved in pancreatic islets formation and insulin synthesis showed that the expression of *Nkx2.2, Ngn3, Isl1, Pdx1, MafA, Nkx6.1,* and insulin was significantly higher in the manipulated group compared with the control group (Fig .4).

### Insulin secretion assay

**Fig.5:**
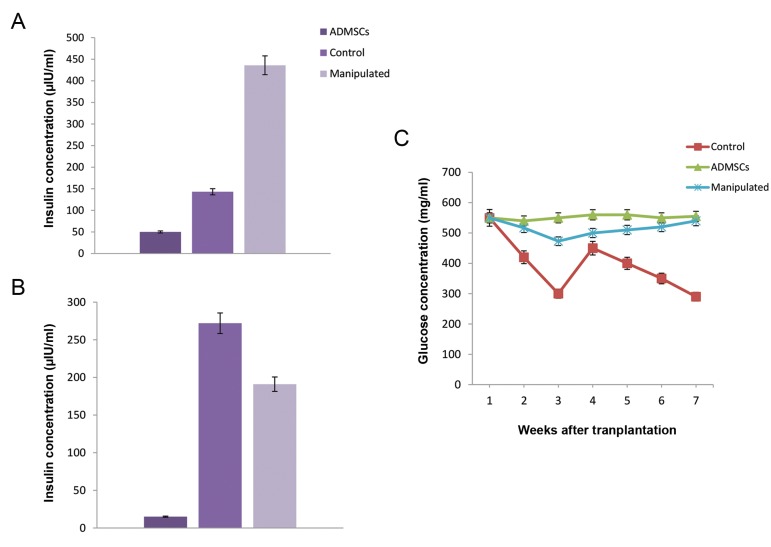
Insulin secretion assay results. **A.** Insulin secretion assay in IPCs beforethe transplantation. MafA+IPCs showed obviously a higher ability of insulinsecretion in comparison to the control IPCs (P<0.05), **B.** Insulin secretion assay in IPCs after the transplantation. The diabetic rats which received thecontrol or MafA+ IPCs showed obviously a higher insulin secretion ability incomparison to un-differentiated ADMSCs (P<0.05), and **C.** The monitoring ofthe blood glucose concentration after the transplantation of IPCs. Diabeticrats that received un-differentiated ADMSCs showed no detectable changein the blood glucose concentration. Rats receiving the positive control IPCs, showed a sharp reduction in the blood glucose concentration within 3 weeksafter the transplantation. After that, the mean blood glucose concentrationraised to 450 mg/dl. Next, the blood glucose concentration was reducedgradually. At sixth week after the transplantation the average amount of glucose concentration reached 290 mg/dl. Diabetic rats receiving themanipulated IPCs or undifferentiated IPCs, showed no detectable ability tocontrol the hyperglycemic condition. IPC; Insulin producing cells and ADMSCs; adipose derived mesenchyal stem cells.

The manipulated group exhibited a significantly higher insulin secretion ability in response to glucose compared with the control group (Fig .5A).

### Evaluation of insulin-producing cells functionality *in vivo*

#### Insulin secretion assay

The measurement of blood insulin concentrations six weeks after transplantation showed significantly higher amounts of the mean rats’ insulin concentration receiving the control and manipulated IPCs compared to rats which received undifferentiated ADMSCs. However, rats receiving the control IPCs secreted the higher amounts of insulin compared to those with manipulated IPCs (Fig .5B). 

### Monitoring of blood glucose concentration 

There was no noticeable difference in the concentration of blood glucose of the STZ-diabetic rats which received undifferentiated ADMSCs during the sixth-week monitoring. When the control IPCs were transplanted to STZ-diabetic rats, a remarkable reduction in the mean blood glucose concentration was observed within 3 weeks. Then, the mean value of blood glucose concentration was gradually elevated. Afterward, the mean value of glucose concentration did not reach the normal glycemic condition until the end of the sixth week after transplantation. There was no obvious reduction in the mean blood glucose concentration in STZ-diabetic rats which were injected by the manipulated IPCs (Fig .5C). 

## Discussion

Recent studies have demonstrated the feasibility of transplanting functional insulin-producing cells which are derived from various sources such as ADMSCs (12, 13, 21). However, some obstacles, such as failure to generate functional IPCs and instability of differentiated cells remain. These problems impede the application of stem cells in the clinical settings (22). 

Treatment with the guidance of homing factors in differentiation of stem cells into IPCs is a suitable way to improve differentiation protocols (23). In this survey, we defined a new protocol for the differentiation of ADMSCs into IPCs using the *MafA* overexpression. In accordance with the previous study, our results showed a successful differentiation of ADMSCs into IPCs (16, 17). The artificial IPCs which were produced in the present study expressed various genes which were related to pancreatic beta cell maturation, maintenance, and insulin secretion including *Nkx2.2, Nkx6.1, Isl-1, Pdx1,* and *Ngn3* (24). Differentiated IPCs exhibited general pancreatic islet cells appearance and ability to secrete insulin in response to glucose exposure (16-18). Then, we overexpressed *MafA* to determine whether this manipulation is capable of promoting the reprogramming potential and insulin production for pancreatic lineage and islet-like characteristics of ADMSCs.

Considering the essential role of *MafA* in the reprogramming of stem cells into pancreatic cells, the maturation of beta cells and maintenance of insulin secretion ability, Matsuoka et al. (25) reported a marked increase in the insulin promoter activity after the overexpression of *MafA*. The main reason for this effect is that *MafA* acts as a transcription factor that binds to a 340 bp promoter region upstream of the transcription start site of the insulin gene (26). 

Therefore, we studied the effect of *MafA* overexpression on the functionality of obtained IPCs. The outcome was an obvious elevation of *Nkx2.2, Ngn3, Isl-1, Pdx1,* and *Nkx6.1* mRNAs expression compared with the control and other experimental groups. Moreover, the insulin expression and secretion were significantly higher in *MafA*+ cells than the control cells. These findings were in accordance with the previous report by Chiou et al. (15) demonstrating that *MafA* promotes the reprogramming of placenta-derived multipotent stem cells into pancreatic islets-like and insulin+ cells. It was also reported that the adenoviral *MafA* overexpression, together with Pdx1 and Ngn3, were markedly induced insulin-producing surrogate cells in pancreatic exocrine cells in adult mice (26). The recent work by Vargas et al. also showed that in the mouse embryo, *MafA* is required at a later time point for the pancreas function and development (27). Taken together, these results revealed a potential for the *MafA* overexpression for the efficient differentiation of stem cells into IPCs in vitro. However, the obtained IPCs were able to secrete insulin, they showed no ability to reduce the blood glucose concentration in diabetic rats (28-30). On the other hand, the amount of secreted insulin was not enough to control the hyperglycemic condition. 

## Conclusion

We have shown that ADMSCs can be effectively differentiated into IPCs through the overexpression of *MafA*. The IPCs obtained via the novel protocol, exhibited the gene expression pattern that mimics pancreatic development, suggesting this in vitro model may be a useful method to induce or increase pancreatic endocrine cell differentiation and may have the potential to be a novel approach for producing ß-islet cells for the cell-based diabetes therapy. The inability of transplanted IPCs in the reduction of hyperglycemia in diabetic rats may originate from an insufficient number of transplanted IPCs or the short-term survival time of the differentiated cells in vivo. Further examinations are required to determine the mechanism by which *MafA* may directly regulate ADMSCs differentiation into IPCs and insulin gene expression. 
